# Background Glucocorticoid Therapy Has No Impact on Efficacy and Safety of Abatacept or Adalimumab in Patients with Rheumatoid Arthritis

**DOI:** 10.3390/jcm9062017

**Published:** 2020-06-26

**Authors:** Yannick Degboé, Michael Schiff, Michael Weinblatt, Roy Fleischmann, Harris A. Ahmad, Arnaud Constantin

**Affiliations:** 1 Rheumatology Centre, Toulouse University Hospital and University Toulouse III Paul Sabatier, 31059 Toulouse, France; constantin.a@chu-toulouse.fr; 2 Rheumatology Division, University of Colorado, Denver, CO 80111, USA; michael.schiff@me.com; 3 Department of Rheumatology & Immunology, Brigham and Women’s Hospital, Boston, MA 02115, USA; mweinblatt@bwh.harvard.edu; 4 Department of Internal Medicine, University of Texas Southwestern Medical Center, Metroplex Clinical Research Center, Dallas, TX 75231, USA; RFleischmann@arthdocs.com; 5 Immunology, Bristol-Myers Squibb, Princeton, NJ 08540, USA; Harris.Ahmad@bms.com

**Keywords:** disease activity, rheumatoid arthritis, biologic DMARDs

## Abstract

To date, the impact of background glucocorticoids (GC) on the efficacy and safety of abatacept or adalimumab in patients with active rheumatoid arthritis (RA) is not clearly established. This post hoc analysis of (AMPLE) trial (NCT00929864) compared efficacy and safety outcomes over 2 years in patients treated with abatacept or adalimumab plus background methotrexate (MTX), who continued GC (≤10 mg/day) versus those who were not receiving GC (no-GC). Of 646 randomized patients, 317 received abatacept + MTX (161 GC, 156 no-GC) and 326 received adalimumab + MTX (162 GC, 164 no-GC). At Year 2, the adjusted mean changes from baseline in Disease Activity Score (DAS28 C-reactive protein (CRP)) and Health Assessment Questionnaire-Disability Index (HAQ-DI) were not significantly different in the GC versus no-GC subgroups receiving abatacept or adalimumab. A similar proportion of patients achieved remission, HAQ-DI score improvement ≥0.3 and radiographic progression rates. No clinically meaningful safety differences were observed between GC versus no-GC subgroups either with abatacept or adalimumab. In patients with active RA of similar baseline disease activity treated with abatacept or adalimumab plus background MTX, there was no additional value of background GC on clinical, functional or radiographic outcomes over two years.

## 1. Introduction

Current guidelines for rheumatoid arthritis (RA) treatment suggest the use of glucocorticoids (GCs) in combination with disease-modifying antirheumatic drugs (DMARDs) at the initiation of therapy, but to use the lowest dose for the shortest period of time necessary [1,2]. However, considerable debate remains regarding dose, timing and duration of use [1,2,3,4]. Although GCs can be effective in decreasing the signs and symptoms of RA, and reducing radiographic progression [2,5,6,7], they may be associated with a range of adverse effects including osteoporosis, hyperglycemia/diabetes mellitus, cardiovascular events and infections [3,5,6].

Abatacept, a T-cell co-stimulation modulator is approved for the treatment of active RA in adults and has been shown to be similarly effective as adalimumab in patients who have active disease despite methotrexate (MTX) therapy [8]. A head-to-head Abatacept versus adaliMumab comparison in biologic-naïve RA subjects with background methotrexate (AMPLE) trial (ClinicalTrials.gov: NCT00929864) showed similar efficacy and tolerability for subcutaneous (SC) abatacept versus the TNF inhibitor adalimumab over two years in patients with active RA and an inadequate response to MTX [8,9]. The trial design allowed enrollment of patients on stable, background GC therapy of ≤10 mg, which had to be maintained at a stable dose for the duration of the two-year study. Patients were not allowed to initiate GCs or reduce or discontinue GCs for reasons other than safety during the study.

Biological (bDMARDs) or targeted synthetic DMARDs (tsDMARDs) randomized controlled trials providing published data about the impact of background GC therapy on clinical and radiological outcomes in RA, are scarce [10,11,12,13]. The objective of this post hoc analysis of the AMPLE trial was to assess whether there was an impact of background GCs at doses of ≤10 mg/day (prednisone equivalent) on the efficacy and safety of SC abatacept or SC adalimumab in biologic-naïve patients with active RA and an inadequate response to MTX. We compared patients with active disease at baseline despite background GCs with those who had similar disease activity but did not receive GCs.

## 2. Experimental Section

### 2.1. Study Design and Analysis Population

The full study design, ethics approvals, study population, inclusion and exclusion criteria and primary results of the AMPLE trial have been described previously [8,9]. Briefly, biologic-naïve patients were randomized 1:1 to receive SC abatacept 125 mg weekly or SC adalimumab 40 mg once every 2 weeks, both in combination with stable MTX for the duration of the trial. In addition, patients were allowed to continue stable, background GCs ≤ 10 mg per day, without change in dose other than for safety reasons.

In AMPLE, all efficacy and safety analyses were performed using the intent-to-treat population, which included all patients who were randomized and received at least one dose of study drug. This post hoc analysis compared clinical, functional and radiographic efficacy outcomes in two subpopulations of patients treated with abatacept plus MTX or adalimumab plus MTX: those who continued stable, background GCs (≤10 mg/day) and those who received no GC therapy (no-GC) during the study.

### 2.2. Ethics Approval

The AMPLE study was approved by the institutional review boards and independent ethics committees at the participating sites (approval of the protocol: 09-5289-0). The study was conducted in accordance with the Declaration of Helsinki and was consistent with the International Conference on Harmonization and Good Clinical Practice.

### 2.3. Outcome Measures

Patient demographics and disease characteristics were analyzed at baseline by treatment and cohort. Clinical, functional and radiographic outcomes were assessed at baseline and at multiple intervals up to Year 2 (Day 729) of the blinded treatment period. Outcomes included the adjusted mean change from baseline in Disease Activity Score in 28 joints (C-reactive protein) (DAS28 (CRP)), Health Assessment Questionnaire-Disability Index (HAQ-DI) and modified total Sharp score (mTSS) at Years 1 and 2. The proportion of patients achieving remission according to Clinical Disease Activity Index (CDAI) and Simplified Disease Activity Index (SDAI) criteria, and the proportion of patients achieving DAS28 (CRP) < 2.6, with improvement in HAQ-DI score ≥ 0.3, and with radiographic nonprogression (mTSS ≤ smallest detectable change (2.2 points) [14]) were also assessed at Years 1 and 2. Safety events were classified using the Medical Dictionary for Regulatory Activities.

### 2.4. Statistical Analysis

All randomized and treated patients were included in the analysis. Baseline patient demographics and disease characteristics were analyzed descriptively by treatment for each cohort. For mean change in DAS28 (CRP) scores, HAQ-DI scores and ACR core component scores, missing values were imputed using a last observation carried forward analysis. For patients who discontinued between years 1 and 2, radiographs were obtained at an early termination visit. In these patients, the 2-year data were imputed using linear extrapolation based on assessments performed at baseline and at the time of discontinuation. Subjects without baseline radiographs were excluded from all radiographic analyses [9]. Adjusted mean changes from baseline in disease activity measures were determined, with corresponding 95% confidence intervals (CIs), at each time point by treatment for each cohort. Endpoints were compared between the cohort of patients who continued background GC therapy and those who were not receiving GC at baseline in each treatment group using a chi-square test for categorical variables and an analysis of covariance model controlling for baseline covariates and DAS28 (CRP) stratification for continuous variables.

## 3. Results

### 3.1. Patient Population

In total, 646 patients were randomly assigned to receive treatment in the AMPLE trial (abatacept, *n* = 318; adalimumab, *n* = 328) [8], of whom 252 abatacept- and 245 adalimumab-treated patients completed Year 2 [9]. The current post hoc analysis included 317/318 abatacept- and 326/328 adalimumab-treated patients. Three patients (1 abatacept and 2 adalimumab) were excluded due to a baseline GC dose > 10 mg. Of the abatacept- and adalimumab-treated patients, 161 and 162, respectively, were on stable background GC at study baseline and 156 and 164, respectively, were not and were not subsequently treated with GC during the study. Baseline demographics and disease activity were similar across all subgroups (Table 1), other than higher baseline mTSS scores in patients on background GC compared with the no-GC subgroup.

### 3.2. Clinical and Functional Evolution

Adjusted mean change (95% CI) from baseline to Years 1 and 2 in DAS28 (CRP) and HAQ-DI are shown in Table A1. There was no difference in improvement (adjusted mean change (95% CI) from baseline) in DAS28 (CRP) between the GC and no-GC subgroups in either the abatacept or adalimumab treatment arms at each time point. The adjusted mean change (95% CI) from baseline in HAQ-DI was significantly greater in the adalimumab/GC subgroup than in the adalimumab/no-GC subgroup at Year 1 (−0.75 (−0.85, −0.65) vs. −0.59 (−0.69, −0.49); *p* = 0.0188), but not at Year 2, and did not differ significantly by GC treatment in abatacept-treated patients.

**Figure 1:**
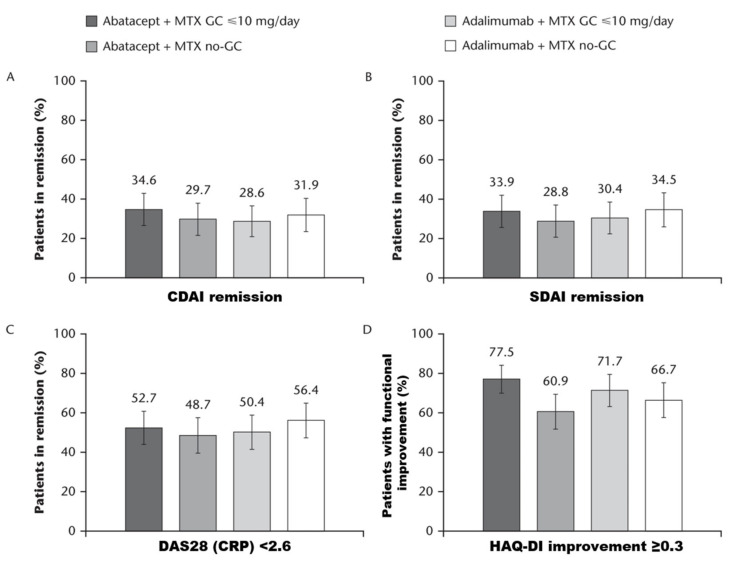
Clinical outcomes at Year 2. Proportion of patients (95% CI) achieving (**A**) CDAI remission, (**B**) SDAI remission, (**C**) DAS28 (CRP) < 2.6, or (**D**) HAQ-DI improvement ≥ 0.3 at Year 2 in abatacept + MTX or adalimumab + MTX GC (≤10 mg/day) and no-GC subgroups. CDAI remission defined as CDAI ≤ 2.8; SDAI remission defined as ≤ 3.3. CDAI: Clinical Disease Activity Index; CI: confidence interval; DAS28 (CRP): Disease Activity Score in 28 joints (C-reactive protein); GC: glucocorticoid; HAQ-DI: Health Assessment Questionnaire-Disability Index; MTX: methotrexate; no-GC: no glucocorticoid; SDAI: Simplified Disease Activity Index.

At Year 2, the proportions of patients in CDAI or SDAI remission or with DAS28 (CRP) < 2.6 or HAQ-DI ≥ 0.3 were similar in the GC and no-GC subgroups for both treatments irrespective of background GC use (Figure 1).

### 3.3. Radiographic Evolution

For both abatacept and adalimumab, there were no statistically significant differences between the background GC and no-GC subgroups either in mean change from baseline in mTSS at Years 1 and 2 (Table 2), or in the proportion of patients without radiographic progression in total Sharp score (TSS; smallest detectable change (SDC) criteria) at Years 1 and 2 (Table A2). There were numerical, but not statistically significant, differences in the proportion of patients without radiographic progression in TSS (SDC criteria) at Years 1 and 2 in favor of the no-GC subgroup (Table A2).

### 3.4. Safety

Adverse events (AEs) during follow-up are shown in Table 3. Two deaths occurred during the study (1 abatacept/no-GC; 1 adalimumab/GC), which were reported previously [8,9].

The abatacept/GC subgroup AE profile was similar compared with the abatacept/no-GC subgroup, with only small numerical, questionably clinically significant reductions in the incidences of major cardiovascular (5.0% vs. 6.4%) and autoimmune (3.1% vs. 4.5%) events, respectively. This was also true in the adalimumab/GC subgroup compared with the adalimumab/no-GC subgroup, with a small, questionably clinically significant numerical increase in the incidence of infectious serious AEs (SAEs) (6.8% vs. 4.9%) and major cardiovascular events (4.9% vs. 3.0%), but with fewer local injection-site reactions (6.8% vs. 14.0%). Although the incidences of malignancies were numerically lower with GC versus no-GC in both treatment groups, the numbers of events were very small.

In the abatacept/GC versus adalimumab/GC subgroups, there was a similar percent of SAEs (13.7% vs. 15.4%), but numerically fewer discontinuations due to SAEs (1.2% vs. 4.9%) and infectious SAEs (3.7% vs. 6.8%), respectively. Similar incidences of major cardiovascular events (5.0% vs. 4.9%) and malignancies (1.9% vs. 1.2%), more autoimmune events (3.1% vs. 1.9%) and fewer local injection-site reactions (4.3% vs. 6.8%) were reported in the abatacept/GC versus adalimumab/GC subgroup, respectively.

In the abatacept/no-GC versus adalimumab/no-GC subgroups, there were numerically fewer discontinuations due to SAEs (1.9% vs. 4.9%) and infectious SAEs (3.8% vs. 4.9%), more autoimmune events (4.5% vs. 1.2%) and fewer local injection-site reactions (3.8% vs. 14.0%).

## 4. Discussion

In biologic-naïve patients with active RA and an inadequate response to MTX who received abatacept or adalimumab, whether patients remained on stable GCs (≤10 mg/day) started prior to baseline, or were not treated with GC during the study, had no detectable impact on clinical, functional or radiographic outcomes over two years. This confirms results of other reports with the same outcomes in the context of tofacitinib, tocilizumab, adalimumab and rituximab treatments [10,11,12,13,15,16,17,18]. In our current study (i) we showed similar results with a different bDMARD, i.e., abatacept, and (ii) we confirmed previous findings about adalimumab. Our observation should be related to the fact that in both groups, whether on background GC or not, patients still had active disease at baseline and the only modification to their therapy was the addition of abatacept or adalimumab, two effective therapies in RA.

A numerical trend toward a higher proportion of patients without radiographic progression in mTSS (SDC criteria) was observed at Years 1 and 2 in favor of the no-GC subgroup. However, these differences were small, unlikely to be clinically significant and may well be accounted for by higher baseline structural disease severity in patients on background GC compared with no-GC.

We identified some slight numerical differences between the GC and no-GC subgroups in terms of safety. However, the relevance of these differences was clinically questionable, except for local injection-site reactions which were less frequent in patients on background GC therapy in the adalimumab group. Given the post hoc nature of the study and the low number of events, the absence of statistical differences may be related to a lack of power. However, these differences are unlikely to be clinically meaningful.

There were significant limitations to this post hoc analysis. The numbers of patients in the GC and no-GC subgroups were small and these patient subgroups were not defined prospectively. Thus, the analysis was not statistically powered to detect differences between the GC and no-GC subgroups. In addition, this trial did not address the effectiveness of adding de novo GCs at ≤10 mg/day to combination therapy with bDMARDs plus MTX in this patient population. Moreover, GCs were not tapered during Year 1 and patients had to remain on stable background GC therapy of ≤10 mg throughout the trial, which is not in accordance with the rapid GC tapering mentioned in the 2019 update of the EULAR recommendations. In a real-life setting, GCs should be rapidly tapered and withdrawn in RA patients with inadequate response to MTX who are clinically and functionally improved after initiation of a bDMARD such as abatacept or adalimumab [2]. For these reasons, we cannot comment on whether there would have been a safety advantage or deleterious clinical consequences of an introduction or reduction of GC dose during the trial.

In RA patients with inadequate response to MTX on concomitant GC and continued active disease, initiating a treatment with abatacept or adalimumab was effective in improving clinical, functional and radiographic outcomes over two years with no meaningful differences from patients without background GC. Consistent with the 2019 EULAR recommendations, we would support GC tapering in RA patients reaching the therapeutic target. A well-designed, properly powered, prospective study is needed to definitively conclude on the efficacy and safety outcomes of low-dose oral GC tapering and discontinuation in patients reaching the therapeutic target under MTX and bDMARD combination.

## Figures and Tables

**Table 1:** Baseline demographics and disease characteristics of patients.

	SC Abatacept + MTX (*n* = 318)		SC Adalimumab + MTX (*n* = 328)	
	GC (*n* = 161)	No-GC (*n* = 156)	GC (*n* = 162)	No-GC (*n* = 164)
Age, years	50.5 (12.7)	52.4 (12.5)	49.2 (12.1)	52.9 (13.0)
Female sex, *n* (%)	136 (84.5)	122 (78.2)	129 (79.6)	139 (84.8)
Disease duration, years	2.0 (1.4)	1.8 (1.4)	2.0 (1.4)	1.5 (1.3)
Physical function (HAQ-DI)	1.6 (0.7)	1.4 (0.6)	1.5 (0.7)	1.4 (0.7)
Number of patients, na	146	148	147	148
mTSS	23.0 (36.6)	15.6 (27.1)	23.1 (62.7)	14.8 (22.4)
ESS	11.9 (19.1)	8.7 (15.9)	12.1 (17.1)	8.9 (13.2)
NSS	11.1 (18.5)	6.9 (12.4)	11.0 (17.6)	6.0 (10.6)
CRP, mg/dL	2.0 (2.6)	1.2 (1.3)	1.5 (1.6)	1.5 (3.7)
DAS28 (CRP)	5.7 (1.1)	5.3 (1.2)	5.6 (1.1)	5.5 (1.1)
MTX dose, mg/week	18.1 (7.9)	16.9 (4.0)	17.2 (3.8)	17.5 (7.9)
GC dose, mg/day	6.6 (2.6)	N/A	6.6 (2.3)	N/A
Anti-CCP2 positive, *n* (%)	70 (43.5)	73 (46.8)	62 (38.3)	91 (55.5)
RF positive, *n* (%)	124 (77.0)	115 (73.7)	133 (82.1)	120 (73.2)

Data from patients included in the AMPLE study (N = 646), by treatment and GC use. Data are mean (SD) unless stated otherwise. GC administered at ≤10 mg/day. All randomized and treated patients were included in the analysis. Number of patients with both baseline and postbaseline X-ray score (van der Heijde modified Sharp scoring system). CCP2: cyclic citrullinated peptide antibody-2; CRP: C-reactive protein; DAS28 (CRP): Disease Activity Score in 28 joints (C-reactive protein); ESS: erosion Sharp score; GC: glucocorticoid; HAQ-DI: Health Assessment Questionnaire-Disability Index; mTSS: modified total Sharp score; MTX: methotrexate; N/A: not applicable; no-GC: no glucocorticoid; NSS: joint space narrowing Sharp score; RF: rheumatoid factor; SC: subcutaneous; SD: standard deviation.

**Table 2:** Change in modified total Sharp score from baseline at Years 1 and 2 ^a^.

	SC Abatacept + MTX (*n* = 318)		SC Adalimumab + MTX (*n* = 328)	
	GC (*n* = 161)	No-GC (*n* = 156)	GC (*n* = 162)	No-GC (*n* = 164)
**Year 1**				
**Total score**				
*n*	146	148	147	148
Baseline mean (SD)	23.02 (36.64)	15.61 (27.11)	23.07 (32.65)	14.83 (22.42)
Mean change from baseline (SD)	0.88 (2.90)	0.24 (2.28)	1.43 (9.06)	0.03 (2.09)
Difference from no-GC (95% CI)	0.67 (0.07, 1.27)	N/A	1.08 (−0.42, 2.58)	N/A
**Erosion score**				
*n*	146	148	147	148
Baseline mean (SD)	11.93 (19.09)	8.67 (15.93)	12.05 (17.11)	8.85 (13.23)
Mean change from baseline (SD)	0.27 (1.87)	0.14 (1.76)	0.57 (5.12)	−0.09 (1.66)
Difference from no-GC (95% CI)	0.17 (−0.24, 0.59)	N/A	0.59 (−0.28, 1.47)	N/A
**Joint space narrowing score**				
*n*	146	148	147	148
Baseline mean (SD)	11.10 (18.54)	6.94 (12.35)	11.02 (17.60)	5.98 (10.58)
Mean change from baseline (SD)	0.62 (2.14)	0.09 (0.95)	0.86 (4.17)	0.12 (0.94)
Difference from no-GC (95% CI)	0.49 (0.11, 0.88)	N/A	0.53 (−0.16, 1.22)	N/A
**Year 2**				
**Total score**				
*n*	130	127	134	124
Baseline mean (SD)	21.56 (33.49)	15.42 (28.25)	22.82 (31.08)	14.00 (21.37)
Mean change from baseline (SD)	1.25 (4.82)	0.52 (3.26)	1.88 (11.51)	0.22 (3.47)
Difference from no-GC (95% CI)	0.75 (−0.27, 1.77)	N/A	1.17 (−0.95, 3.29)	N/A
**Erosion score**				
*n*	130	127	134	124
Baseline mean (SD)	11.30 (17.03)	8.81 (16.77)	12.09 (17.22)	8.43 (12.14)
Mean change from baseline (SD)	0.49 (2.56)	0.32 (2.58)	0.75 (6.65)	−0.01 (2.26)
Difference from no-GC (95% CI)	0.21 (−0.42, 0.84)	N/A	0.63 (−0.61, 1.87)	N/A
**Joint space narrowing score**				
*n*	130	127	134	124
Baseline mean (SD)	10.26 (17.31)	6.61 (12.53)	10.74 (15.80)	5.58 (10.52)
Mean change from baseline (SD)	0.75 (2.87)	0.20 (1.05)	1.13 (5.05)	0.23 (1.51)
Difference from no-GC (95% CI)	0.53 (−0.01, 1.07)	N/A	0.60 (−0.32, 1.53)	N/A

^a^ All randomized and treated patients. GC administered at ≤10 mg/day. CI: confidence interval; GC: glucocorticoid; MTX: methotrexate; N/A: not applicable; no-GC: no glucocorticoid; SC: subcutaneous; SD: standard deviation.

**Table 3:** Summary of patients with adverse events reported over 2 years.

**System Organ Class, *n* (%)**	**SC Abatacept + MTX (*n* = 318)**		***p* Value**	**SC Adalimumab + MTX (*n* = 328)**		***p* Value**
	**GC (*n* = 161)**	**No-GC (*n* = 156)**		**GC (*n* = 162)**	**No-GC (*n* = 164)**	
Deaths	0	1 (0.6)	0.492	1 (0.6)	0	0.497
SAEs	22 (13.7)	22 (14.1)	0.999	25 (15.4)	29 (17.7)	0.656
Discontinued due to SAEs	2 (1.2)	3 (1.9)	0.681	8 (4.9)	8 (4.9)	1.000
Infectious SAEs	6 (3.7)	6 (3.8)	1.000	11 (6.8)	8 (4.9)	0.488
Major cardiovascular events	8 (5.0)	10 (6.4)	0.633	8 (4.9)	5 (3.0)	0.412
Malignancies	3 (1.9)	4 (2.6)	0.720	2 (1.2)	5 (3.0)	0.448
Autoimmune events ^a^	5 (3.1)	7 (4.5)	0.569	3 (1.9)	2 (1.2)	0.684
Local injection-site reactions	7 (4.3)	6 (3.8)	1.000	11 (6.8)	23 (14.0)	0.045

GC administered at ≤10 mg/day. ^a^ Excludes new autoantibody status (e.g., double-stranded DNA autoantibody positivity). GC: glucocorticoid; MTX: methotrexate; no-GC: no glucocorticoid; SAE: serious adverse event; SC: subcutaneous. *p* value from Fisher exact test comparing GC vs. no-GC group in each arm.

## Appendix A

**Table A1:** Adjusted mean change from baseline in DAS28 (CRP) and HAQ-DI at Years 1 and 2 ^a^.

	SC Abatacept + MTX (*n* = 318)		SC Adalimumab + MTX (*n* = 328)	
	GC (*n* = 161)	No-GC (*n* = 156)	GC (*n* = 162)	No-GC (*n* = 164)
**DAS28 (CRP)**				
Year 1, *n*	142	132	138	128
Adjusted mean change from baseline (95% CI)	−2.52 (−2.73, −2.31)	−2.37 (−2.58, −2.16)	−2.59 (−2.80, −2.38)	−2.50 (−2.71, −2.29)
Estimate of difference (95% CI)	−0.14 (−0.43, 0.14)	N/A	−0.09 (−0.38, 0.19)	N/A
Year 2, *n*	131	119	125	117
Adjusted mean change from baseline (95% CI)	−2.75 (−2.99, −2.52)	−2.55 (−2.79, −2.31)	−2.59 (−2.82, −2.35)	−2.62 (−2.86, −2.39)
Estimate of difference (95% CI)	−0.20 (−0.52, 0.12)	N/A	0.04 (−0.28, 0.36)	N/A
**HAQ-DI**				
Year 1, *n*	139	128	135	126
Adjusted mean change from baseline (95% CI)	−0.72 (−0.83, −0.62)	−0.62 (−0.72, −0.51)	−0.75 (−0.85, −0.65)	−0.59 (−0.69, −0.49)
Estimate of difference (95% CI)	−0.11 (−0.25, 0.04)	N/A	−0.17 (−0.30, −0.03)	N/A
Year 2, *n*	129	115	120	108
Adjusted mean change from baseline (95% CI)	−0.74 (−0.85, −0.62)	−0.64 (−0.75, −0.53)	−0.73 (−0.84, −0.62)	−0.61 (−0.72, −0.50)
Estimate of difference (95% CI)	−0.10 (−0.25, 0.06)	N/A	−0.12 (−0.27, 0.03)	N/A

^a^ All randomized and treated patients. GC administered at ≤10 mg/day. CI: confidence interval; DAS28 (CRP): Disease Activity Score in 28 joints (C-reactive protein); GC: glucocorticoid; HAQ-DI: Health Assessment Questionnaire-Disability Index; MTX: methotrexate; N/A: not applicable; no-GC: no glucocorticoid; SC: subcutaneous.

**Table A2:** Proportion of patients without radiographic progression in total Sharp score at Years 1 and 2 ^a^.

Change from Baseline ≤ SDC (2.2)	SC Abatacept + MTX (*n* = 318)		SC Adalimumab + MTX (*n* = 328)	
	GC (*n* = 161)	No-GC (*n* = 156)	GC (*n* = 162)	No-GC (*n* = 164)
Year 1				
Number of patients, *n*/N (%)	123/146 (84.25)	135/148 (91.22)	125/147 (85.03)	137/148 (92.57)
95% CI	78.34, 90.16	86.66, 95.78	79.27, 90.80	88.34, 96.79
Estimate of difference (95% CI)	−7.0 (−15.1, 1.2)	N/A	−7.5 (−15.4, 0.3)	N/A
Year 2				
Number of patients, n/N (%)	105/130 (80.77)	113/127 (88.98)	107/134 (79.85)	110/124 (88.71)
95% CI	73.99, 87.54	83.53, 94.42	73.06, 86.64	83.14, 94.28
Estimate of difference (95% CI)	−8.2 (−17.7, 1.3)	N/A	−8.9 (−18.4, 0.7)	N/A

Progression in total Sharp score definition is based on the smallest detectable change (SDC) criterion. ^a^ All randomized and treated patients. GC administered at ≤10 mg/day. CI: confidence interval; GC: glucocorticoid; N: number of patients with both baseline and postbaseline X-ray total score based on imputed data; MTX: methotrexate; N/A: not applicable; no-GC: no glucocorticoid; SC: subcutaneous; SDC: smallest detectable change.
